# Supplementary material for: A population-based cohort study of socio-demographic risk factors for COVID-19 deaths in Sweden
Source: Nat Commun. 2020 Oct 9;11:5097. doi: 10.1038/s41467-020-18926-3 (PMC7547672; doi:10.1038/s41467-020-18926-3)
Supplement: Supplementary file 1 — Supplementary Information [file 41467_2020_18926_MOESM1_ESM.pdf]

A population-based cohort study of socio-demographic risk factors for COVID-19 deaths in Sweden

Supplementary Information

Corresponding author: Sven Drefahl
email: sven.drefahl@sociology.su.se

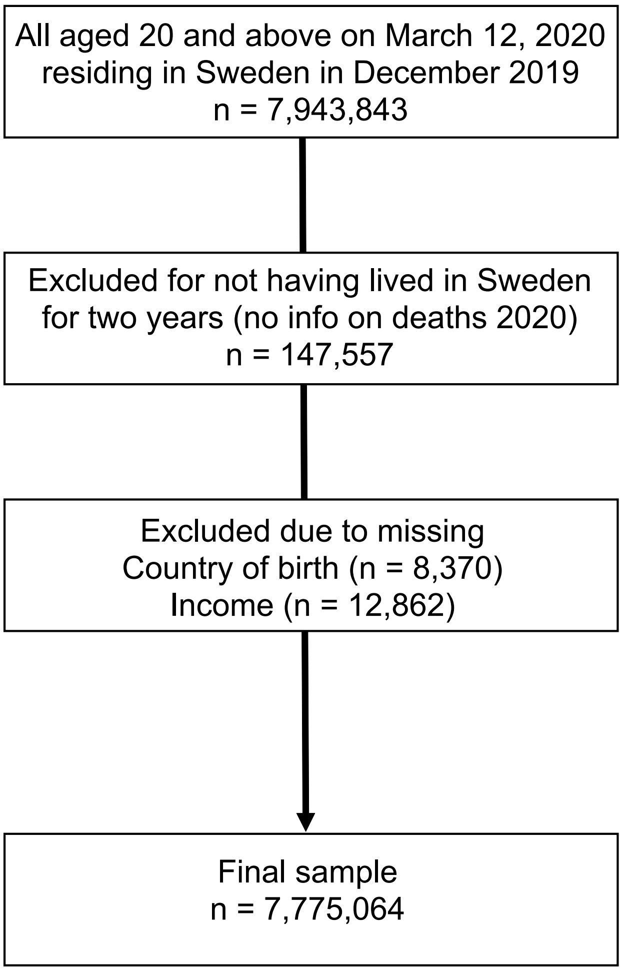

Supplementary Fig. 1. **Flow diagram showing the inclusion and exclusion criteria.**

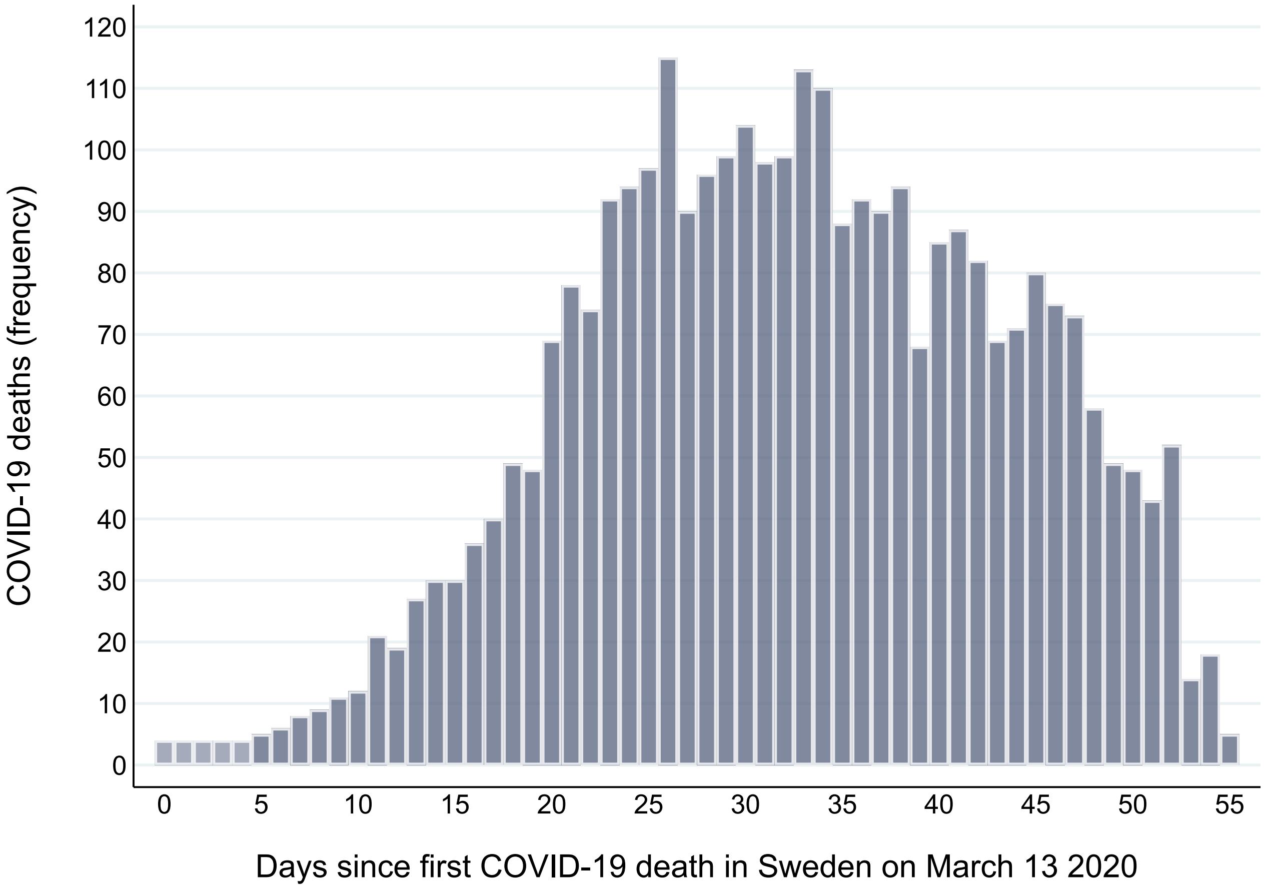

Supplementary Fig. 2. **COVID-19 deaths reported up to May 7, 2020 by days since first COVID-19 death in Sweden on March 13, 2020.** n = 3,126. Days with less than 5 deaths are set to a value of 4 in order to meet the confidentiality requirements of the data provider.

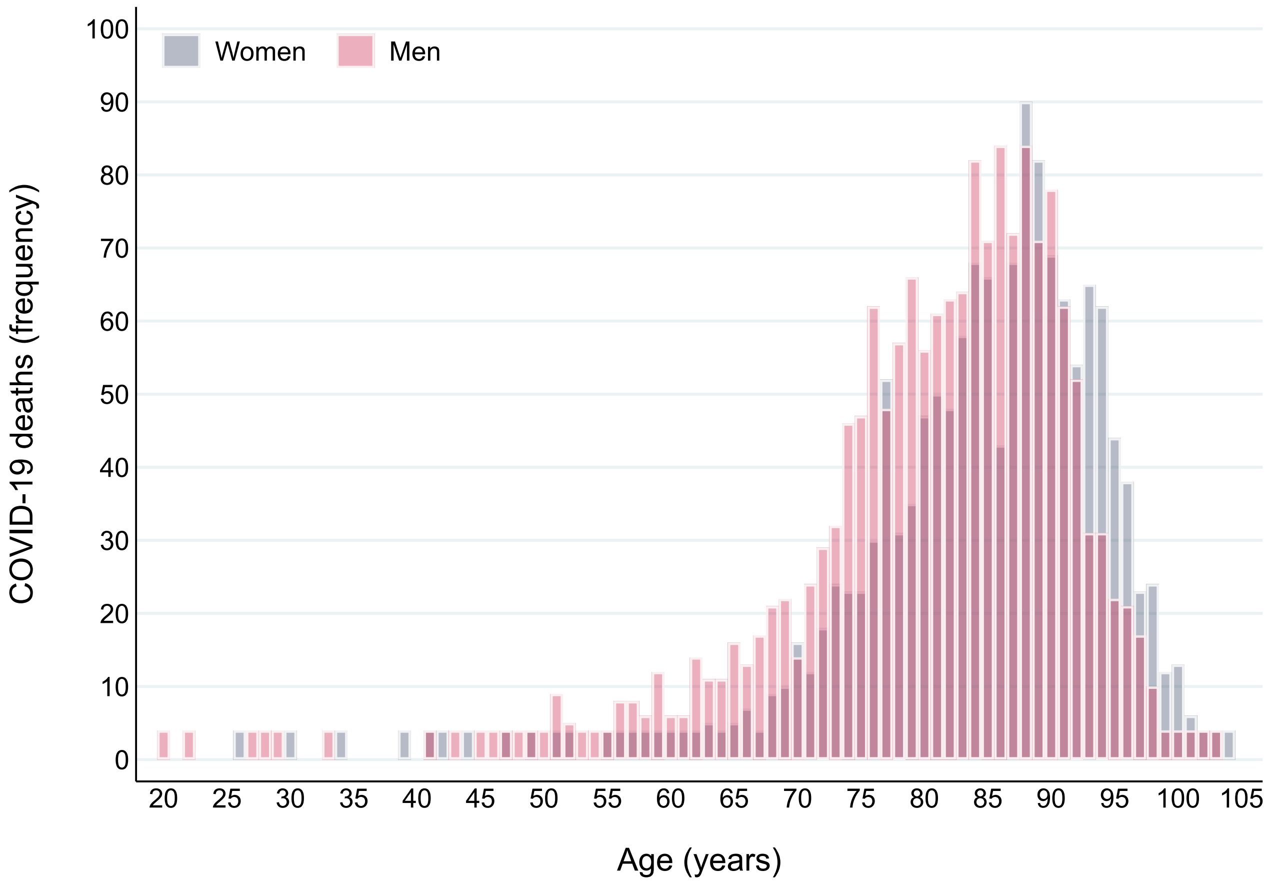

Supplementary Fig. 3. **COVID-19 deaths by single age and sex in Sweden between March 13, 2020 and May 7, 2020.** n = 3,126. Single ages with less than 5 deaths are set to a value of 4 in order to meet the confidentiality requirements of the data provider.

Supplementary Table 1. Deaths and Exposure time to the risk at death from COVID-19 by age group for women in Sweden (March 13, 2020 – May 7, 2020)

	Age Group									
	20-49		50-69		70-79		80-89		90+	
	D	Exp	D	Exp	D	Exp	D	Exp	D	Exp
Civil status										
Married	5	102,242	24	96,219	74	40,405	130	11,757	26	892
Never married	8	159,973	15	39,909	35	7,331	53	2,045	26	506
Divorced	<5	22,730	16	39,319	90	16,734	124	6,328	64	1,063
Widowed	<5	906	10	7,013	65	14,192	313	18,608	364	8,245
Education										
Primary	<5	27,589	22	22,630	95	20,131	262	16,250	237	5,896
Secondary	5	111,777	26	85,527	109	34,106	217	13,977	161	3,188
Post-Second	<5	140,845	11	73,070	47	23,489	104	7,770	64	1,353
Missing	<5	5,640	6	1,233	13	934	37	741	18	269
Individual net income										
Tertile 1 (Low)	10	95,238	45	46,480	204	50,438	475	30,037	341	8,268
Tertile 2	<5	114,198	17	70,624	41	19,904	108	6,810	100	1,901
Tertile 3 (High)	<5	76,416	<5	65,356	19	8,320	37	1,891	39	536
Country of birth										
Sweden	11	215,889	46	146,393	198	68,566	475	33,476	407	9,509
HIC	<5	15,523	5	14,277	36	6,953	85	3,944	55	952
LMIC other	<5	36,396	10	14,864	19	2,281	33	955	8	177
LMIC MENA	<5	18,044	<5	6,926	11	861	27	363	10	68
County of residence										
Other	7	213,505	40	142,919	119	63,551	305	31,954	236	8,698
Stockholm	7	72,347	25	39,540	145	15,110	315	6,784	244	2,008
Total[a]	14	285,852	65	182,460	264	78,662	620	38,738	480	10,706

D Deaths *Exp* Exposure time to the risk of dying from COVID-19 *HIC* high-income countries *LMIC MENA l*ow-middle income countries from Northern Africa and the Middle East *LMIC other* other low-middle income countries. Number of deaths below 5 cannot be reported to in order to meet the confidentiality requirements of the data provider. [a] Sum of exposure time over all categories may not always add up to the total because of rounding.

Supplementary Table 2. Deaths and Exposure time to the risk at death from COVID-19 by age group for men in Sweden (March 13, 2020 – May 7, 2020)

	Age Group									
	20-49		50-69		70-79		80-89		90+	
	D	Exp	D	Exp	D	Exp	D	Exp	D	Exp
Civil status										
Married	<5	90,056	100	98,811	211	46,269	324	17,351	135	1,971
Never married	12	195,284	59	50,475	69	9,697	56	2,068	15	278
Divorced	<5	16,090	38	32,592	105	12,845	130	4,014	29	391
Widowed	0	261	<5	2,246	40	4,934	198	5,381	156	2,054
Education										
Primary	<5	38,689	61	31,335	148	22,152	286	11,851	108	2,154
Secondary	7	145,039	99	90,754	168	30,555	256	10,351	135	1,546
Post-Second	<5	108,784	34	60,461	90	20,269	143	6,184	79	896
Missing	5	9179	5	1,573	19	768	23	428	13	99
Individual net income										
Tertile 1 (Low)	9	86,000	103	35,288	236	27,305	349	13,706	144	2,547
Tertile 2	5	91,411	49	50,519	130	28,600	282	11,590	158	1,669
Tertile 3 (High)	<5	124,280	47	98,317	59	17,839	77	3,519	33	479
Country of birth										
Sweden	8	227,825	110	149,881	315	64,866	545	25,348	291	4,257
HIC	<5	16,973	16	12,831	42	5,591	89	2,466	31	334
LMIC other	5	34,525	37	12,012	36	2,156	40	716	6	69
LMIC MENA	0	22,368	36	9,399	32	1,132	34	285	7	35
County of residence										
Other	7	226,989	82	144,679	216	60,509	373	23,998	184	3,889
Stockholm	9	74,702	117	39,444	209	13,236	335	4,816	151	806
Total[a]	16	301,691	199	184,123	425	73,745	708	28,814	335	4,695

D Deaths *Exp* Exposure time to the risk of dying from COVID-19 *HIC* high-income countries *LMIC MENA l*ow-middle income countries from Northern Africa and the Middle East *LMIC other* other low-middle income countries. Number of deaths below 5 cannot be reported to in order to meet the confidentiality requirements of the data provider. [a] Sum of exposure time over all categories may not always add up to the total because of rounding.

Supplementary Table 3: Hazard ratios of dying from COVID-19 and all other causes of death for men and women in Sweden

		COVID-19		All other causes of death	
Variables	Characteristics	HR (95% CI)	P value	HR (95% CI)	P value
Men					
Civil Status	Married (ref.)	1.00		1.00	
	Never Married	1.49 (1.28–1.75)	<0.001	1.57 (1.46–1.68)	<0.001
	Divorced	1.42 (1.25–1.63)	<0.001	1.51 (1.41–1.61)	<0.001
	Widowed	1.52 (1.33–1.73)	<0.001	1.42 (1.32–1.52)	<0.001
Education Level	Primary	1.24 (1.07–1.43)	0.003	1.22 (1.14–1.31)	<0.001
	Secondary	1.25 (1.09–1.43)	0.001	1.11 (1.04–1.19)	0.002
	Post-Second (ref.)	1.00		1.00	
Individual net income	Tertile 1 (Low)	1.76 (1.49–2.09)	<0.001	1.93 (1.78–2.09)	<0.001
	Tertile 2	1.51 (1.29–1.78)	<0.001	1.44 (1.33–1.56)	<0.001
	Tertile 3 (ref.)	1.00		1.00	
Country of birth	Sweden (ref.)	1.00		1.00	
	HIC	1.19 (1.01–1.39)	0.034	0.96 (0.88–1.05)	0.359
	LMIC other	2.20 (1.81–2.69)	<0.001	0.92 (0.81–1.06)	0.245
	LMIC MENA	3.13 (2.51–3.90)	<0.001	0.82 (0.68–0.99)	0.035
County of residence	Other (ref.)	1.00		1.00	
	Stockholm	4.51 (4.08–5.00)	<0.001	1.24 (1.16–1.31)	<0.001
Women					
Civil status	Married (ref.)	1.00		1.00	
	Never Married	2.05 (1.66–2.53)	<0.001	1.71 (1.56–1.87)	<0.001
	Divorced	1.81 (1.53–2.14)	<0.001	1.46 (1.35–1.58)	<0.001
	Widowed	1.54 (1.32–1.80)	<0.001	1.38 (1.29–1.48)	<0.001
Education level	Primary	1.51 (1.28–1.79)	<0.001	1.45 (1.34–1.56)	<0.001
	Secondary	1.38 (1.17–1.62)	<0.001	1.30 (1.20–1.40)	<0.001
	Post-Second (ref.)	1.00		1.00	
Individual net income	Tertile 1 (Low)	1.26 (1.01–1.58)	0.044	1.60 (1.44–1.78)	<0.001
	Tertile 2	0.99 (0.78–1.25)	0.921	1.22 (1.09–1.36)	<0.001
	Tertile 3 (ref.)	1.00		1.00	
Country of birth	Sweden (ref.)	1.00		1.00	
	HIC	1.08 (0.92–1.26)	0.361	0.96 (0.89–1.05)	0.382
	LMIC other	1.45 (1.12–1.90)	0.006	0.91 (0.78–1.06)	0.212
	LMIC MENA	2.09 (1.52–2.89)	<0.001	0.81 (0.64–1.03)	0.088
County of residence	Other (ref.)	1.00		1.00	
	Stockholm	4.69 (4.21–5.23)	<0.001	1.11 (1.05–1.19)	<0.001

Hazard ratios (HRs) and 95% confidence intervals (CIs) were calculated with the use of multivariate Cox proportional hazard models for men (n = 3,876,881) and women (n = 3,898,173) separately. All models are adjusted for age (baseline). Two-sided P values were calculated from Cox proportional hazard models. *HIC* high-income countries *LMIC MENA l*ow-middle income countries from Northern Africa and the Middle East *LMIC other* other low-middle income countries. Variable for Education level also includes missing category.

Supplementary Table 4: Hazard ratios of dying from COVID-19 and all other causes of death in Sweden for ages below 66 and ages 66 and older

		COVID-19		All other causes of death	
		HR (95% CI)	P value	HR (95% CI)	P value
Ages<66					
Sex	Women (ref.)	1.00		1.00	
	Men	2.97 (2.14–4.13)	<0.001	1.84 (1.65–2.04)	<0.001
Civil status	Married (ref.)	1.00		1.00	
	Never Married	1.48 (1.04–2.10)	0.029	1.58 (1.40–1.80)	<0.001
	Divorced	0.93 (0.63–1.39)	0.738	1.58 (1.37–1.81)	<0.001
	Widowed	1.19 (0.43–3.29)	0.736	1.71 (1.19–2.45)	0.003
Education level	Primary	2.62 (1.65–4.16)	<0.001	1.94 (1.66–2.25)	<0.001
	Secondary	2.22 (1.46–3.37)	<0.001	1.33 (1.17–1.51)	<0.001
	Post-Second (ref.)	1.00		1.00	
Individual net income	Tertile 1 (Low)	5.40 (3.51–8.35)	<0.001	4.00 (3.49–4.58)	<0.001
	Tertile 2	2.11 (1.32–3.36)	0.002	1.56 (1.35–1.81)	<0.001
	Tertile 3 (ref.)	1.00		1.00	
County of birth	Sweden (ref.)	1.00		1.00	
	HIC	1.03 (0.60–1.77)	0.904	0.68 (0.55–0.84)	<0.001
	LMIC other	2.48 (1.67–3.68)	<0.001	0.67 (0.55–0.81)	<0.001
	LMIC MENA	2.31 (1.47–3.63)	<0.001	0.52 (0.40–0.67)	<0.001
County of residence	Other (ref.)	1.00		1.00	
	Stockholm	3.86 (2.88–5.16)	<0.001	1.12 (0.99–1.26)	0.079
Ages>=66					
Sex	Women (ref.)	1.00		1.00	
	Men	2.01 (1.86–2.18)	<0.001	1.61 (1.55–1.67)	<0.001
Civil status	Married (ref.)	1.00		1.00	
	Never Married	1.65 (1.44–1.89)	<0.001	1.58 (1.49–1.69)	<0.001
	Divorced	1.62 (1.46–1.80)	<0.001	1.46 (1.39–1.54)	<0.001
	Widowed	1.48 (1.34–1.63)	<0.001	1.37 (1.31–1.44)	<0.001
Education level	Primary	1.30 (1.16–1.45)	<0.001	1.30 (1.23–1.37)	<0.001
	Secondary	1.26 (1.13–1.40)	<0.001	1.19 (1.12–1.25)	<0.001
	Post-Second (ref.)	1.00		1.00	
Individual net income	Tertile 1 (Low)	1.35 (1.18–1.55)	<0.001	1.45 (1.35–1.55)	<0.001
	Tertile 2	1.17 (1.02–1.34)	0.027	1.18 (1.10–1.27)	<0.001
	Tertile 3 (ref.)	1.00		1.00	
County of birth	Sweden (ref.)	1.00		1.00	
	HIC	1.14 (1.02–1.28)	0.025	0.99 (0.93–1.06)	0.868
	LMIC other	1.78 (1.49–2.12)	<0.001	0.99 (0.88–1.11)	0.890
	LMIC MENA	2.90 (2.38–3.55)	<0.001	0.96 (0.80–1.14)	0.614
County of residence	Other (ref.)	1.00		1.00	
	Stockholm	3.90 (2.91–5.22)	<0.001	1.16 (1.11–1.21)	<0.001

Hazard ratios (HRs) and 95% confidence intervals (CIs) were calculated with the use of multivariate Cox proportional hazard models for ages<66 (n = 5,813,359) and ages>=66 (n = 1,979,710) separately. All models are adjusted for age (baseline). Two-sided P values were calculated from Cox proportional hazard models. *HIC* high-income countries *LMIC MENA l*ow-middle income countries from Northern Africa and the Middle East *LMIC other* other low-middle income countries. Variable for Education level also includes missing category.

Supplementary Table 5: Hazard ratios of dying from COVID-19 in Sweden – Univariate Cox regression & Multivariate Cox regression of the total study population

		Univariate (age-adjusted)		Multivariate	
		HR (95% CI)	P value	HR (95% CI)	P value
Sex	Women (ref.)	1.00		1.00	
	Men	1.73 (1.61–1.86)	<0.001	2.09 (1.93–2.26)	<0.001
Civil status	Married (ref.)	1.00		1.00	
	Never Married	1.61 (1.42–1.82)	<0.001	1.66 (1.46–1.88)	<0.001
	Divorced	1.54 (1.39–1.71)	<0.001	1.57 (1.41–1.73)	<0.001
	Widowed	1.11 (1.01–1.22)	0.023	1.48 (1.34–1.63)	<0.001
Education level	Primary	1.15 (1.04–1.27)	0.007	1.34 (1.20–1.49)	<0.001
	Secondary	1.22 (1.10–1.35)	<0.001	1.30 (1.17–1.44)	<0.001
	Post-Second (ref.)	1.00		1.00	
Individual net income	Tertile 1 (Low)	1.22 (1.08–1.39)	0.002	1.60 (1.40–1.83)	<0.001
	Tertile 2	1.26 (1.11–1.44)	<0.001	1.32 (1.16–1.51)	<0.001
	Tertile 3 (ref.)	1.00		1.00	
County of birth	Sweden (ref.)	1.00		1.00	
	HIC	1.52 (1.36–1.70)	<0.001	1.14 (1.02–1.28)	0.022
	LMIC other	2.76 (2.38–3.19)	<0.001	1.90 (1.62–2.22)	<0.001
	LMIC MENA	5.06 (4.30–5.95)	<0.001	2.77 (2.31–3.32)	<0.001
County of residence	Other (ref.)	1.00		1.00	
	Stockholm	4.50 (4.20–4.83)	<0.001	4.63 (4.30–4.99)	<0.001

Hazard ratios (HRs) and 95% confidence intervals (CIs) were calculated with the use of multivariate Cox proportional hazard models. All models are adjusted for age (baseline). Two-sided P values were calculated from Cox proportional hazard models. *HIC* high-income countries *LMIC MENA l*ow-middle income countries from Northern Africa and the Middle East *LMIC other* other low-middle income countries. Variable for Education level also includes missing category.

Supplementary Table 6. Hazard ratios of dying from all other causes of deaths in Sweden – Univariate Cox regression adjusted for age & Multivariate Cox regression of the total study population

		Univariate (age-adjusted)		Multivariate	
		HR (95% CI)	P value	HR (95% CI)	P value
Sex	Women (ref.)	1.00		1.00	
	Men	1.43 (1.39–1.48)	<0.001	1.67 (1.61–1.73)	<0.001
Civil status	Married (ref.)	1.00		1.00	
	Never Married	1.71 (1.62–1.81)	<0.001	1.62 (1.53–1.71)	<0.001
	Divorced	1.43 (1.36–1.50)	<0.001	1.49 (1.42–1.56)	<0.001
	Widowed	1.21 (1.16–1.27)	<0.001	1.40 (1.34–1.47)	<0.001
Education level	Primary	1.57 (1.50–1.65)	<0.001	1.32 (1.25–1.39)	<0.001
	Secondary	1.35 (1.29–1.42)	<0.001	1.19 (1.13–1.25)	<0.001
	Post-Second (ref.)	1.00		1.00	
Individual net income	Tertile 1 (Low)	1.71 (1.61–1.82)	<0.001	1.79 (1.68–1.91)	<0.001
	Tertile 2	1.37 (1.29–1.46)	<0.001	1.35 (1.27–1.44)	<0.001
	Tertile 3 (ref.)	1.00		1.00	
County of birth	Sweden (ref.)	1.00		1.00	
	HIC	1.01 (0.96–1.08)	0.627	0.96 (0.91–1.02)	0.228
	LMIC other	1.04 (0.95–1.15)	0.382	0.92 (0.84–1.02)	0.121
	LMIC MENA	1.00 (0.87–1.15)	0.978	0.83 (0.72–0.96)	0.013
County of residence	Other (ref.)	1.00		1.00	
	Stockholm	1.06 (1.01–1.10)	0.009	1.18 (1.13–1.23)	<0.001

Hazard ratios (HRs) and 95% confidence intervals (CIs) were calculated with the use of multivariate Cox proportional hazard models. All models are adjusted for age (baseline). Two-sided P values were calculated from Cox proportional hazard models. *HIC* high-income countries *LMIC MENA l*ow-middle income countries from Northern Africa and the Middle East *LMIC other* other low-middle income countries. Variable for Education level also includes missing category.

Supplementary Table 7. Odds-Ratios of dying from COVID-19 for men and women in Sweden

		Men		Women	
Variables	Characteristics	OR (95% CI)	P value	OR (95% CI)	P value
Age	20–29	0.01 (0.00–0.02)	<0.001	0.01 (0.00–0.03)	<0.001
	30–34	0.00 (0.00–0.03)	<0.001	0.01 (0.00–0.06)	<0.001
	35–39	0		0.02 (0.00–0.07)	<0.001
	40–44	0.03 (0.01–0.05)	<0.001	0.04 (0.02–0.11)	<0.001
	45–49	0.04 (0.02–0.08)	<0.001	0.03 (0.01–0.08)	<0.001
	50–54	0.12 (0.08–0.18)	<0.001	0.03 (0.01–0.09)	<0.001
	55–59	0.19 (0.13–0.27)	<0.001	0.06 (0.03–0.14)	<0.001
	60–64	0.30 (0.22–0.41)	<0.001	0.21 (0.13–0.34)	<0.001
	65–69	0.58 (0.44–0.75)	<0.001	0.38 (0.26–0.56)	<0.001
	70–74 (ref.)	1.00		1.00	
	75–79	2.36 (1.93–2.88)	<0.001	2.10 (1.63–2.70)	<0.001
	80–84	4.63 (3.80–5.63)	<0.001	4.82 (3.81–6.09)	<0.001
	85–89	10.22 (8.42–12.41)	<0.001	8.83 (6.99–11.16)	<0.001
	90–94	16.41 (13.27–20.29)	<0.001	13.86 (10.88–17.65)	<0.001
	95–99	19.96 (14.87–26.79)	<0.001	18.58 (14.09–24.50)	<0.001
	100+	17.22 (6.96–42.62)	<0.001	27.24 (17.41–42.62)	<0.001
Civil Status	Married (ref.)	1.00		1.00	
	Never Married	1.46 (1.24–1.71)	<0.001	2.07 (1.68–2.56)	<0.001
	Divorced	1.41 (1.23–1.61)	<0.001	1.84 (1.55–2.18)	<0.001
	Widowed	1.57 (1.38–1.79)	<0.001	1.62 (1.39–1.90)	<0.001
Education Level	Primary	1.24 (1.08–1.43)	0.003	1.54 (1.30–1.82)	<0.001
	Secondary	1.25 (1.09–1.42)	0.001	1.38 (1.17–1.63)	<0.001
	Post-Second (ref.)	1.00		1.00	
Individual net income	Tertile 1 (Low)	1.82 (1.54–2.16)	<0.001	1.29 (1.03–1.61)	0.027
	Tertile 2	1.55 (1.32–1.83)	<0.001	1.00 (0.79–1.27)	0.995
	Tertile 3 (ref.)	1.00		1.00	
Country of birth	Sweden (ref.)	1.00		1.00	
	HIC	1.19 (1.01–1.39)	0.035	1.08 (0.92–1.26)	0.369
	LMIC other	2.18 (1.79–2.66)	<0.001	1.42 (1.09–1.85)	0.010
	LMIC MENA	3.07 (2.46–3.84)	<0.001	2.01 (1.46–2.78)	<0.001
County of residence	Other (ref.)	1.00		1.00	
	Stockholm	4.55 (4.11–5.04)	<0.001	4.74 (4.25–5.29)	<0.001

Odds ratios (ORs) and 95% confidence intervals (CIs) were calculated with the use of multivariate logistic regression models. Two-sided P values were calculated from logistic regression models. *HIC* high-income countries *LMIC MENA l*ow-middle income countries from Northern Africa and the Middle East *LMIC other* other low-middle income countries. Variable for Education level also includes missing category.
