# Supplementary material for: A population-based cohort study of socio-demographic risk factors for COVID-19 deaths in Sweden
Source: Nat Commun. 2020 Oct 9;11:5097. doi: 10.1038/s41467-020-18926-3 (PMC7547672; doi:10.1038/s41467-020-18926-3)
Supplement: Supplementary file 3 — Reporting Summary [file 41467_2020_18926_MOESM3_ESM.pdf]

nature research

Corresponding author(s): Sven Drefahl
Last updated by author(s): Sep 10, 2020

Reporting Summary

Nature Research wishes to improve the reproducibility of the work that we publish. This form provides structure for consistency and transparency in reporting. For further information on Nature Research policies, see our Editorial Policies and the Editorial Policy Checklist.

Statistics

For all statistical analyses, confirm that the following items are present in the figure legend, table legend, main text, or Methods section.

n/a	Confirmed	
☐	☒	The exact sample size (n) for each experimental group/condition, given as a discrete number and unit of measurement
☐	☒	A statement on whether measurements were taken from distinct samples or whether the same sample was measured repeatedly
☐	☒	The statistical test(s) used AND whether they are one- or two-sided<br>*Only common tests should be described solely by name; describe more complex techniques in the Methods section.*
☐	☒	A description of all covariates tested
☒	☐	A description of any assumptions or corrections, such as tests of normality and adjustment for multiple comparisons
☐	☒	A full description of the statistical parameters including central tendency (e.g. means) or other basic estimates (e.g. regression coefficient) AND variation (e.g. standard deviation) or associated estimates of uncertainty (e.g. confidence intervals)
☐	☒	For null hypothesis testing, the test statistic (e.g. F, t, r) with confidence intervals, effect sizes, degrees of freedom and P value noted<br>*Give P values as exact values whenever suitable.*
☒	☐	For Bayesian analysis, information on the choice of priors and Markov chain Monte Carlo settings
☒	☐	For hierarchical and complex designs, identification of the appropriate level for tests and full reporting of outcomes
☒	☐	Estimates of effect sizes (e.g. Cohen's d, Pearson's r), indicating how they were calculated

Our web collection on statistics for biologists contains articles on many of the points above.

Software and code

Policy information about availability of computer code

Data collection	We use administratively collected register data of the total Swedish population. The data collected in different Swedish authorities were linked through the unique personal identification number at Statistics Sweden. The sole responsibility for data collection lies within Statistics Sweden. The authors used no special software for data collection.
Data analysis	The authors access the individual-level data through Statistics Sweden’s micro-online access system MONA. All data analyses were done with STATA 16.0

For manuscripts utilizing custom algorithms or software that are central to the research but not yet described in published literature, software must be made available to editors and reviewers. We strongly encourage code deposition in a community repository (e.g. GitHub). See the Nature Research guidelines for submitting code & software for further information.

Data

Policy information about availability of data

All manuscripts must include a data availability statement. This statement should provide the following information, where applicable:

- Accession codes, unique identifiers, or web links for publicly available datasets
- A list of figures that have associated raw data
- A description of any restrictions on data availability

This study is produced under the Swedish Statistics Act, where privacy concerns restrict the availability of register data for research. Aggregated data can be made available by the authors, conditional on ethical vetting. The authors access the individual-level data through Statistics Sweden’s micro-online access system MONA. The authors linked data from the "Historical Population Register (HBR)", the "Register of the Total Population (RTB)", the "Cause of Death Register", and the "Longitudinal integrated database for health insurance and labour market studies (LISA)". More information about data availability and data access can be obtained from: https://www.scb.se/en/services/guidance-for-researchers-and-universities/mona--a-system-for-delivering-microdata/. The analyses have been approved by

the Swedish ethical-vetting authority, Dnr 2020-02199.

Field-specific reporting

Please select the one below that is the best fit for your research. If you are not sure, read the appropriate sections before making your selection.

☐ Life sciences ☒ Behavioural & social sciences ☐ Ecological, evolutionary & environmental sciences

For a reference copy of the document with all sections, see nature.com/documents/nr-reporting-summary-flat.pdf

Behavioural & social sciences study design

All studies must disclose on these points even when the disclosure is negative.

Study description	Quantitative longitudinal study of COVID-19 mortality in Sweden.
Research sample	Total Swedish population aged 20 and above on March 12, 2020. All deaths from COVID-19 and other causes of death in Sweden reported between March 13 - May 7, 2020.
Sampling strategy	No sampling, the study includes the total population of Sweden.
Data collection	We use administratively collected register data of the total Swedish population. The data collected in different Swedish authorities were linked through the unique personal identification number at Statistics Sweden. The sole responsibility for data collection lies within Statistics Sweden.
Timing	Individual are observed from March 12, 2020 to May 7, 2020.
Data exclusions	Data was administratively collected by Swedish authorities. Data from these authorities were linked by Statistics Sweden through a unique personal identifier. We excluded individuals who had not lived in Sweden in the two prior years (N=147,557) because those could not be linked to all records of data. We also excluded a small percentage of individuals for whom we had no registered information on country of birth (N=8,370) and income (N=12,862). The final study population consists of 7,775,064 individuals amounting to a total of 1,189,484 person-years under observation.
Non-participation	As we study administrative data of the total Swedish population, no study subjects have the possibility to decline participation.
Randomization	No experiments were used in this study. No randomization was performed as the study population includes the total population.

Reporting for specific materials, systems and methods

We require information from authors about some types of materials, experimental systems and methods used in many studies. Here, indicate whether each material, system or method listed is relevant to your study. If you are not sure if a list item applies to your research, read the appropriate section before selecting a response.

Materials & experimental systems

n/a	Involved in the study	
☒	☐	Antibodies
☒	☐	Eukaryotic cell lines
☒	☐	Palaeontology and archaeology
☒	☐	Animals and other organisms
☐	☒	Human research participants
☒	☐	Clinical data
☒	☐	Dual use research of concern

Methods

n/a	Involved in the study	
☒	☐	ChIP-seq
☒	☐	Flow cytometry
☒	☐	MRI-based neuroimaging

Human research participants

Policy information about studies involving human research participants

Population characteristics	See above
Recruitment	No selective recruitment, the total Swedish population aged 20 and above was included.
Ethics oversight	The analyses have been approved by the Swedish ethical-vetting authority, Dnr 2020-02199.

Note that full information on the approval of the study protocol must also be provided in the manuscript.
